# Exploring Social Support and Quality of Life Among Mothers of Children with Autism Spectrum Disorders: A Cross-Sectional Study

**DOI:** 10.3390/healthcare13020095

**Published:** 2025-01-07

**Authors:** Khulud Ahmad Rezq, Haia Mahdi Hindi Albalawi, Hanan F. Alharbi

**Affiliations:** 1 Community & Mental Health Nursing Department, Faculty of Nursing, University of Tabuk, Tabuk 47512, Saudi Arabia; 2 Maternal & Child Health Nursing Department, Faculty of Nursing, University of Tabuk, Tabuk 47512, Saudi Arabia; h.malbalawi@ut.edu.sa; 3 Maternity & Pediatrics Nursing Department, Faculty of Nursing, Princess Nourah bint Abdulrahman University, Riyadh 11564, Saudi Arabia; hfalharbi@pnu.edu.sa

**Keywords:** autism spectrum disorder, social support, quality of life, mothers, psychological stress

## Abstract

**Background**: Mothers of children with autism spectrum disorder (ASD) often experience significant stress, which can adversely affect their quality of life (QoL) and increase their reliance on social support. This study aimed to explore the relationship between social support and QoL among mothers of ASD children and identify associated factors. **Methods**: A cross-sectional study was conducted from December 2022 to March 2023, involving 218 mothers of ASD children in Saudi Arabia. An online questionnaire was distributed via autism associations. Inclusion criteria were mothers of children under 18 diagnosed with ASD, excluding those with diagnosed mental illnesses. Social support and QoL were measured using the Multidimensional Scale of Perceived Social Support (MSPSS) and the Quality of Life in Autism Questionnaire (QoLA). Statistical analysis was performed using Jamovi software. **Results**: The mean MSPSS and QoLA scores were 4.87 and 100.88, respectively, with a significant positive correlation (Spearman’s rho = 0.509, *p* < 0.001). Social support was positively associated with higher education and negatively with having more than one autistic child. QoLA scores were significantly predicted by family income (>SAR 10,000 or US 2667) and MSPSS score (*p* < 0.001). **Conclusions**: Social support enhances maternal QoL and is influenced by educational level and income, highlighting the need for targeted interventions to support mothers with multiple ASD children. While individual support is important, prioritizing societal accessibility may offer more effective long-term solutions by proactively addressing systemic challenges faced by autistic individuals and their families.

## 1. Introduction

Autism Spectrum Disorder (ASD) is a neurodevelopmental condition that affects approximately 1 in 100 children worldwide [1]. It is characterized by social isolation, repetitive behaviors, challenges in sensory processing, and intense, focused interests [2,3]. Co-occurring conditions, such as Attention Deficit Hyperactivity Disorder (ADHD) and intellectual disabilities, are often associated with ASD, further amplifying the challenges of raising a child with this condition [4]. These comorbidities demand that parents dedicate significant time, financial resources, emotional resilience, and physical energy to meet their children’s needs [5,6].

Raising a child with ASD presents lifelong challenges. Many parents report difficulties in managing these demands, as they often prioritize the needs of their autistic child above all else, which can strain the family dynamic [7]. Moreover, this leads to an increase in the financial cost of the family to provide for all their needs, which increases stress and reduces the parents’ quality of life [8,9,10].

Mothers, as primary caregivers, spend substantial amounts of time attending to their autistic children. Unlike mothers in the general population, whose caregiving time typically decreases as their children enter school, the mothers of autistic children maintain stable caregiving demands during this stage [11,12,13]. On average, their caregiving time can reach six and a half hours daily, compared to only three hours for mothers in the general population [14]. This increased caregiving burden leads to heightened perceptions of time pressure, adversely affecting mothers’ social engagement, community participation, QoL, and employment opportunities [11,14,15]. The need to support parents of autistic children is critical, as adequate social support can significantly enhance their QoL and contribute to the effective rehabilitation of their children [16]. Studies have shown that social support acts as a protective factor, reducing autism severity, improving rehabilitation outcomes [17,18], increasing parental satisfaction, and alleviating stress and pressure [2,19].

In Saudi Arabia, the prevalence of Autism Spectrum Disorder ASD is notably high at 2.51% compared to Qatar and Lebanon with rates of 1.14% and 1.50%, respectively. In contrast, Oman and Bahrain report much lower rates, possibly reflecting underdiagnosis or limited healthcare access. The Middle East and North Africa (MENA) region’s overall pooled prevalence of 0.13% is significantly lower than the global average of 1%, highlighting the need for better diagnostic practices and increased ASD awareness [20]. Although the Saudi government provides resources and funding for families with ASD children, these services often fall short of addressing all their needs [21]. Cultural norms and the intensive training required for effective intervention exceeding 30 h per week pose additional challenges for parents [21]. Studies conducted in Saudi Arabia have explored the impact of ASD on caregivers’ QoL. A cross-sectional study conducted in Makkah City involving 812 caregivers found that those raising children with ASD had significantly lower QoL scores compared to caregivers of neurotypical children. Influential factors included caregiver age, relationship to the child, residence, and access to medical services [22]. In Tabuk City, a study of 100 parents reported that caregiving responsibilities most strongly affected the psychological and environmental domains of QoL. Sociodemographic factors, such as marital status, income, and the duration of the child’s illness, were found to play significant roles in determining QoL outcomes [23]. Similarly, in Arar City, a study involving 84 parents revealed that 63.1% of caregivers experienced impaired QoL. Key factors contributing to this included gender, unemployment, low income, and the the child’s illness duration [24]. Therefore, our primary aim is to determine the social support levels and QoL among mothers raising autistic children, while the secondary objectives are to determine the correlation between both QoL and social support, as well as their associated factors.

## 2. Materials and Methods

### 2.1. Research Design and Setting

We performed a cross-sectional study including mothers of autistic children in the Saudi population to investigate their QoL and social support levels in addition to the determining factors associated with them. This study was conducted from December 2022 to March 2023 by communicating with autism associations in Saudi Arabia and sending an online questionnaire to be shared with mothers of autistic children.

This study was conducted in Saudi Arabia, a rapidly growing economy and a diverse population of approximately 36 million with a growth domestic product (GDP) growth rate of 2.8% [25]. The Saudi healthcare system is undergoing significant modernization, focusing on improving access and quality of care. The Vision 2030 reform initiative aims to enhance healthcare services for all citizens, including those with disabilities [26]. Notably, the Saudi government has implemented various initiatives to support individuals with autism and their families, such as establishing specialized centers and developing inclusive education programs [27].

### 2.2. Respondents and Sampling

A purposive sampling technique was used to collect the study data by reaching the autism associations in all regions of Saudi Arabia. The minimum sample size was determined using G-Power 3.1 software [28]. The minimum number required for this study was 180. However, the sample size was increased by 20% to compensate for possible participants not meeting the inclusion criteria; therefore, the required total sample size was 216. We included mothers to autistic children aged less than 18 years old, while we excluded mothers who were diagnosed with any mental illnesses, whether they were on treatment or not.

### 2.3. Instruments and Data Collection

Data were collected through online questionnaires by communicating with autism associations in Saudi Arabia and sending an online questionnaire to be shared with the mothers of autistic children. The questionnaires were composed of three parts: (1) participants’ baseline and demographic characteristics, including maternal age, marital status, occupational status, nationality, educational level, number of children, family income, and residency region; (2) a Multidimensional Scale of Perceived Social Support (MSPSS) score which measures social support levels; and a Quality of Life in Autism questionnaire (QolA) score which measures the maternal QoL.

The MSPSS score was generated in 1988 to measure the perception of social support among parents with autistic children. It comprises three main categories: friend support, family support, and other kinds of support. The questionnaire comprises 12 questions graded by a seven-point Likert scale ranging from very strongly disagree with a score of one point to very strongly agree with a score of seven. The scale alpha coefficient is 0.93 [29]. The score can be categorized by calculating the mean score into three groups: low social support with mean scores ranging from 1 to 2.9, moderate social support with mean scores ranging from 3 to 5, and high social support with mean scores ranging from 5.1 to 7 [30].

The QoLA score is a 28-item questionnaire generated in 2014 to assess the QoL of parents raising autistic children. It covers many aspects like financial status, community support, health satisfaction, negative psychological impact, and self-satisfaction. By increasing the score, a high QoL is concluded. The alpha coefficient of the QoLA score is 0.92 [31]. Our primary outcome was to describe the social support and QoL levels in mothers with autistic children, while our secondary outcomes were to determine the correlation between both variables, as well as the factors associated with them.

### 2.4. Statistical Analysis

We conducted the analysis using Jamovi software (version 2.4.8.0). Numbers and frequencies present qualitative variables, while continuous variables are presented in median with an interquartile range or mean with standard deviation. The normality of the data was tested using the Shapiro–Wilk test, which showed that our data were not normally distributed. Therefore, we used non-parametric statistical tests to compare variables like the Kruskal–Wallis H test in the case of ordinal or continuous variables and the Chi-square test in the case of qualitative variables. Spearman’s correlation analysis was used to determine the correlation between MSPSS and QoLA scores. Finally, we performed univariate and multivariate linear regression analyses to determine the predictors of the MSPSS and QoLA scores. Results were significant when *p* values were less than 0.05.

## 3. Results

**Figure 1:**
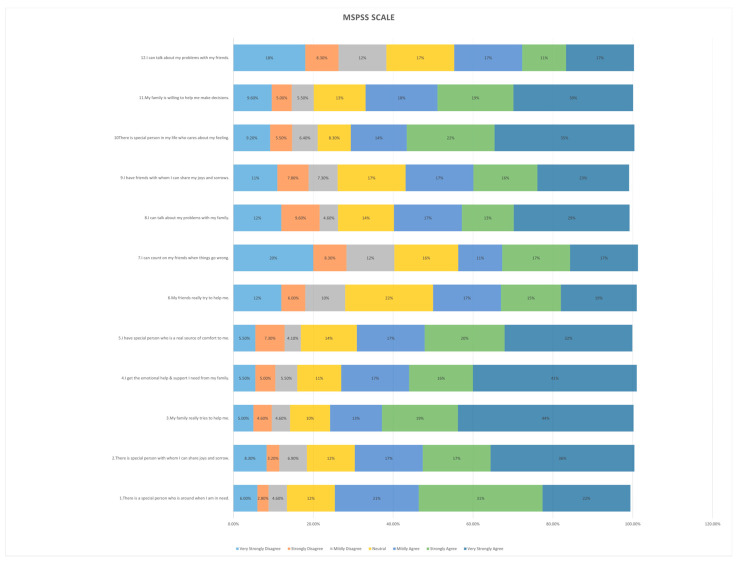
Distribution of responses to the Multidimensional Scale of Perceived Social Support (MSPSS) items among mothers of children with Autism Spectrum Disorder.

**Figure 2:**
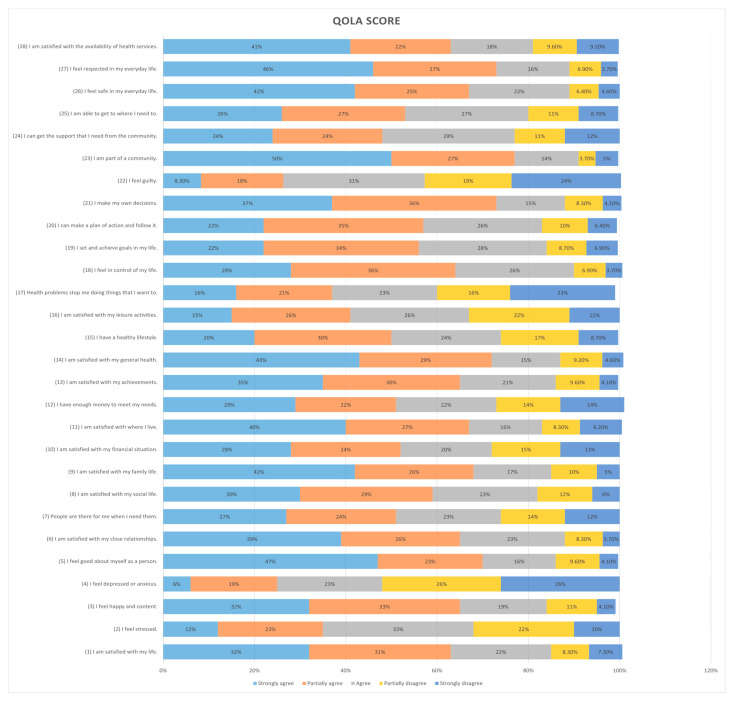
Distribution of responses to the Quality of Life in Autism (QoLA) questionnaire items among mothers of children with Autism Spectrum Disorder.

Our study included 218 mothers to autistic children, most of them were aged from 30 to 39 years old (40.4%), were married (89.9%), were non-employed (57.3%), were Saudi (92.2%), had a bachelor’s degree (52.8%), had more than three children (37.2%), had at least one autistic child (94.5%), were living in the northern region (62.8%), and hada family income between SAR 5000 and SAR 10,000 per month (42.2%), Table 1 shows the full details of the baseline and demographic characteristics of the study population. The mean total MSPSS score of the participants was 4.87, while the total QolA score mean was 100.88; the full details of THE MSPSS and QolA questionnaire results are presented in Figure 1 and Figure 2.

**Figure 3:**
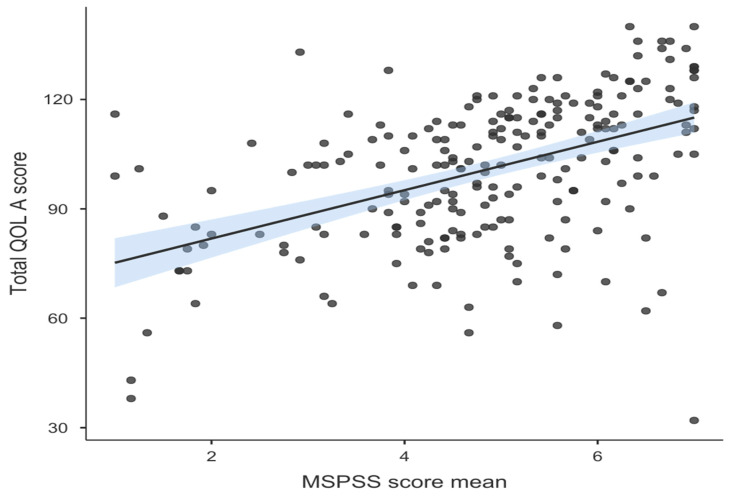
Correlation between MSPSS and QoLA scores.

We performed a subgroup analysis according to the social support by classifying the participants into those who receive high social support with mean scores ranging from 5.1 to 7 (111 mothers), moderate social support with mean scores ranging from 3 to 5 (84 mothers), or low social support with mean scores ranging from 1 to 2.9 (23 mothers). The high social support was significantly the highest in women with one child (57.1%) and women who have one autistic child (52.9%), with *p* values of 0.04 and <0.001, respectively. The QoLA mean score was significantly increased in women who received high social support (5.67) compared to moderate and low social support (4.22 and 1.91, respectively) in the other two groups, *p* < 0.001; Table 2 shows the full details of the social support subgroup analysis. We also found a significant positive correlation between the MSPSS score and QoLA score (Spearman’s rho = 0.509 and *p* < 0.001), Figure 3.

We investigated the predictors of the MSPSS score by performing univariate and multivariate regression analyses. We found that the MSPSS score had a significant positive association in the univariate model with maternal ages between 20 and 29 years (*p* = 0.046), postgraduate degrees (*p* = 0.006), family incomes between SAR 5000 and SAR 10,000 (*p* = 0.022), family income with more than SAR 10,000 (*p* = 0.012), and an increased QoLA score (*p* < 0.001). In contrast, it had a significant negative association with a number of autistic children (*p* < 0.001). In multivariate regression analysis, the MSPSS score had positive significant associations with high school certificates (*p* = 0.016), postgraduate degrees (*p* = 0.008), and QolA scores (*p* < 0.001). In contrast, it had a significant negative association with the number of autistic children (*p* < 0.001); Table 3 shows the full details.

In the univariate analysis of factors associated with the QoLA score, we found significant positive association with family incomes between SAR 5000 and SAR 10,000 (*p* = 0.016), family incomes of more than SAR 10,000 (*p* < 0.001), and the MSPSS score (*p* < 0.001), while in the multivariate regression analysis, only family incomes of more than SAR 10,000 (*p* = 0.014) and MSPSS score (*p* < 0.001) had significant positive associations; Table 4 shows the full details.

## 4. Discussion

Our study measured the social support levels and QoL levels in 218 mothers of autistic children and found that most had above-average QolA scores of 100.88 and most had high social support levels (50.9%). We found that the high social support was significantly the highest in women with one child (57.1%) and women who have one autistic child (52.9%). Also, high social support had significantly the highest QoLA scores, which was confirmed by the positive correlation between both scores. We found that high educational levels, the number of autistic children, and the QoLA score were predictors of social support, while family income and the MSPSS score were the predictors of maternal QoL.

We conducted our study in only mothers as they are more prone to stress, anxiety, and depression compared to fathers, and QoL scores are decreased in autistic children’s mothers, especially compared to fathers [32,33], as females are associated with lower QoL scores regarding social functioning, bodily pain, and physical health compared to males [34]. Also, most autistic children’s caregivers are usually mothers, and most of them are susceptible to stress more than fathers [35,36].

We found that social support and QoL were positively correlated, and positive associations were found in univariate and multivariate regression analyses. This was supported by many cross-sectional studies [34,37,38,39]. Also, a meta-analysis found that social support was positively associated with QoL [40]. Social support means to provide a sense of care, respect, help, and love through social networks [41]. Therefore, increasing levels of social support can lead to decreased stress levels [39,42]. Also, it decreases depression levels, improves well-being [43], and decreases inner pain [44].

These findings are essential to improve social support for families with ASD children by providing awareness campaigns to decrease social stigma [45], in addition to educating healthcare workers about the importance of providing social support to families with autistic children [46].

Socioeconomic disparities and quality of life are further affected by cultural influences. Kang-Yi et al. (2018) observed differences in support systems between collectivist and individualist cultures, noting that families in collectivist societies, such as many in Asia, often rely on community and religious networks. In contrast, families in individualist cultures, common in Western countries, frequently value independence and privacy as key components of well-being [47]. This cultural variation suggests that parental perceptions of quality of life are likely shaped by their cultural background. Moreover, it raises the possibility that the concept of quality of life may hold different meanings across cultures, potentially necessitating culturally sensitive approaches to support and intervention. Therefore, improving the quality of life for families experiencing challenges requires not only considering material supports like financial aid and educational resources but also recognizing and respecting the diverse cultural frameworks through which well-being is understood and experienced [48].

Family income affects mental health, vitality, and social involvement [34]. Also, many studies showed that increasing family income was associated with improved QoL levels [49,50,51], as decreased income was associated with decreased self-esteem, personal control, aspiration, and efficacy [52]. All these findings were in line with our results of the improvement of the QoLA scores with incomes of more than SAR 10,000 (about USD 2667) in multivariate regression analysis; however, we found only a significant association between increased family income and the increased social support score in univariate regression analysis without multivariate regression analysis; therefore, family income could not be associated with social support, which requires more studies to confirm these findings.

We found that educational level was associated with social support; however, it was not associated with QoLA scores. Published studies found that parent’s education level did not affect the QoL [49,53]. On the other hand, many studies found that educational level was associated with improved QoL [54,55,56]. While limited data supported our findings on the association between social support and educational levels in the literature, more studies are needed to confirm our findings and solve this controversy. We also found a significant negative association between the number of autistic children and social support, which was not deeply investigated in the literature; however, we could explain it by increasing autistic children in the family; the children requirement increased, which in turn increased maternal overwhelm and stress, decreased the time of social involvement, and increased the demand of social support.

Our study highlighted the importance of social support in improving mothers’ QoL who are raising children with ASD and studied the factors associated with QoL and social support. Identifying factors like family income, educational level, and number of autistic children can target management plans and identify individuals who are at risk of poor social support and QoL. Therefore, the health stakeholders should provide awareness campaigns about the nature of the disorder to prevent social stigma, perform educating programs for the caregivers to educate them about dealing with autistic children in addition to their families, and improve the socioeconomic status of their families, as this can lead to an increase in their income which in turn improves QoL.

**Strengths and limitations:** the main strength is that we performed cross-sectional studies, including many autism centers in all regions of Saudi Arabia, and investigated many factors that affect the QoL and social support of the mothers of autistic children. However, the main limitation is that we cannot conclude our results regarding causality as our study is a cross-sectional study. Therefore, longitudinal studies with large follow-up periods are needed to support our findings. Also, larger sample-sized studies, including all varieties of socioeconomic levels, are needed to confirm our results. A key limitation of this study is the reliance on self-reported data from mothers regarding both their own experiences and their children’s autism diagnoses, which were not independently verified, potentially impacting the accuracy and generalizability of our findings. In addition, the lack of detailed child-related data, such as age, severity of ASD, educational placement, and behavioral interventions, is a limitation of the findings of this study. The absence of these variables may have impacted our ability to fully understand the complex relationship between child characteristics and parental QoL. However, previous studies have reported that no statistical significance was observed in terms of quality of life and the level of global developmental delay or gestational age of ASD children, the school attendance of the ASD child, the severity of the disorder, nor the ability of the ASD child to speak 10 words [45,53]. However, more factors need to be studied. Thus, we recommend future studies that incorporate a more comprehensive assessment of the child’s condition to further investigate these important factors and their impact on parental well-being.

## 5. Conclusions

Social support was essential to improve maternal QoL. Social support had a positive association with mothers’ educational level and QoL, while it had a negative association with an increased number of autistic children. The QoLA score was positively associated with social support and family incomes of more than SAR 10,000 (USD 2667). Despite the fact that social support is important, shifting the focus from individual support to societal accessibility might offer more effective long-term solutions. Creating universally accessible environments could potentially lessen the need for individualized interventions by proactively addressing many of the challenges faced by autistic individuals and their families.

## Figures and Tables

**Table 1:** The baseline characteristics of the participants. MSPSS = Multidimensional Scale of Perceived Social Support, QolA = Quality of Life A scale, SD = Standard Deviation, IQR = interquartile range.

Variable (*n* = 218)	Counts	% of Total
Maternal age		
Less than 20 years	5	2.3%
20–29 years	60	27.5%
30–39 years	88	40.4%
More than 40 years	65	29.8%
Marital status		
Married	196	89.9%
Divorced or separated	21	9.6%
Widow	1	0.5%
Occupational status		
Employed	93	42.7%
Non-employed	125	57.3%
Nationality		
Saudi	201	92.2%
Non-Saudi	17	7.8%
Educational level		
Less than high school education	30	13.8%
High school certificate	59	27.1%
Bachelor degree	115	52.8%
Postgraduate degree	14	6.4%
Number of children		
One	28	12.8%
Two	53	24.3%
Three	56	25.7%
More than three	81	37.2%
Number of autistic children		
One	206	94.5%
More than one	12	5.5%
Family income		
Less than SAR 5000	46	21.1%
SAR 5000–10,000	92	42.2%
More than SAR 10,000	80	36.7%
Residency region		
Central	27	12.4%
Western	33	15.1%
Southern	12	5.5%
Eastern	9	4.1%
Northern	137	62.8%
MSPSS score		
Mean (SD)	4.87	1.44
Median (IQR)	5	(4.17, 6)
MSPSS categories		
High support	111	0.509
Moderate support	84	0.385
Low support	23	0.106
Significant other subscale		
Mean (SD)	5.19	1.53
Median (IQR)	5.5	(4.31, 6.25)
Family subscale		
Mean (SD)	5.16	1.64
Median (IQR)	5.5	(4.31, 6.5)
Friends’ subscale		
Mean (SD)	4.25	1.77
Median (IQR)	4.38	(3, 5.5)
Total QoL A score		
Mean (SD)	100.88	19.84
Median (IQR)	103	(86.25, 115)

**Table 2:** Subgroup analysis according to social support. MSPSS = Multidimensional Scale of Perceived Social Support, QolA = Quality of Life A scale, SD = Standard Deviation, IQR = Interquartile range, ***n*** = number.

Variable	MSPSS Categories						Total	*p*
	High Support *n* = 111		Moderate Support *n* = 84		Low Support *n* = 23			
	*n*	%	*n*	%	*n*	%		
Marital status								
Married	103	52.6%	73	37.2%	20	10.2%	196	0.523
Divorced or separated	8	38.1%	10	47.6%	3	14.3%	21	
Widow	0	0.0%	1	100.0%	0	0.0%	1	
Occupational status								
Employed	42	45.2%	43	46.20%	8	8.60%	93	0.126
Non-employed	69	55.2%	41	32.80%	15	12%	125	
Nationality								
Saudi	101	50.2%	79	39.30%	21	10.40%	201	0.722
Non-Saudi	10	58.8%	5	29.40%	2	11.80%	17	
Number of autistic children								
One	109	52.9%	79	38.30%	18	8.70%	206	<0.001
More than one	2	16.7%	5	41.70%	5	41.70%	12	
Residency region								
Central	12	44.4%	11	40.70%	4	14.80%	27	0.125
Western	14	42.4%	12	36.40%	7	21.20%	33	
Southern	7	58.3%	3	25%	2	16.70%	12	
Eastern	2	22.2%	5	55.60%	2	22.20%	9	
Northern	76	55.5%	53	38.70%	8	5.80%	137	
Total	111	50.9%	84	38.50%	23	10.60%	218	
Maternal age								
Less than 20 years	1	20.0%	2	40.0%	2	40.0%	5	0.084
20–29 years	37	61.7%	17	28.3%	6	10.0%	60	
30–39 years	41	46.6%	35	39.8%	12	13.6%	88	
More than 40 years	32	49.2%	30	46.2%	3	4.6%	65	
Educational level								
Less than high school education	11	36.7%	15	50.0%	4	13.3%	30	0.637
High school certificate	34	57.6%	21	35.6%	4	6.8%	59	
Bachelor degree	57	49.6%	44	38.3%	14	12.2%	115	
Postgraduate degree	9	64.3%	4	28.6%	1	7.1%	14	
Number of children								
One	16	57.1%	7	25.0%	5	17.9%	28	0.04
Two	26	49.1%	19	35.8%	8	15.1%	53	
Three	27	48.2%	23	41.1%	6	10.7%	56	
More than three	42	51.9%	35	43.2%	4	4.9%	81	
Family income								
Less than SAR 5000	19	41.3%	17	37.0%	10	21.7%	46	0.097
SAR 5000–10,000	46	50.0%	40	43.5%	6	6.5%	92	
More than SAR 10,000	46	57.5%	27	33.8%	7	8.8%	80	
QoLA score								
Mean (SD)	5.67	0.6	4.22	0.531	1.91	0.632		<0.001
Median (IQR)	6	(5.42, 6.42)	4.33	(3.9, 4.6)	1.83	(1.42, 2.46)		

**Table 3:** The univariate and multivariate regression analyses of factors associated with the MSPSS score.

Predictor	Univariate Regression Analysis				Multivariate Regression Analysis			
	Estimate	95% Confidence Interval		*p*	Estimate	95% Confidence Interval		*p*
		Lower	Upper			Lower	Upper	
Maternal age:								
20–29 years—Less than 20 years	1.326	0.024	2.63	0.046	0.0825	−1.1294	1.2943	0.893
30–39 years—Less than 20 years	0.824	−0.4619	2.11	0.208	−0.3808	−1.5972	0.8356	0.538
More than 40 years—Less than 20 years	1.182	−0.1165	2.48	0.074	0.0748	−1.127	1.2765	0.902
Marital status:								
Divorced or separated—Married	−0.444	−1.09	0.205	0.179	-	-	-	-
Widow—Married	−1.332	−4.16	1.499	0.355	-	-	-	-
Occupational status:								
Non-employed—Employed	−0.0757	−0.464	0.312	0.701	-	-	-	-
Nationality:								
Non-Saudi—Saudi	0.261	−0.454	0.976	0.472	-	-	-	-
Educational level:								
High school certificate—Less than high school education	0.577	−0.0483	1.203	0.07	0.667	0.1274	1.2066	0.016
Bachelor’s degree—Less than high school education	0.298	−0.2738	0.87	0.305	0.3384	−0.1722	0.8489	0.193
Postgraduate degree—Less than high school education	1.266	0.363	2.169	0.006	1.0982	0.2954	1.9011	0.008
A number of children:								
Two—One	−0.195	−0.857	0.468	0.563	-	-	-	-
Three—One	−0.116	−0.772	0.54	0.728	-	-	-	-
More than three—One	0.13	−0.492	0.752	0.681	-	-	-	-
The number of autistic children:								
More than one—One	−1.59	−2.41	−0.779	<0.001	−1.4667	−2.2309	−0.7025	<0.001
Family income:								
SAR 5000–10,000—Less than SAR 5000	0.589	0.084	1.09	0.022	0.3306	−0.1101	0.7713	0.141
More than SAR 10,000—Less than SAR 5000	0.668	0.1512	1.19	0.012	0.2392	−0.2327	0.7111	0.319
Residency region:								
Western—Central	−0.242	−0.972	0.488	0.515	-	-	-	-
Southern—Central	0.116	−0.86	1.092	0.815	-	-	-	-
Eastern—Central	−0.778	−1.86	0.305	0.158	-	-	-	-
Northern—Central	0.226	−0.366	0.819	0.452	-	-	-	-
Total QOL A score	0.0347	0.0262	0.0433	<0.001	0.031	0.0225	0.0395	<0.001

**Table 4:** The univariate and multivariate regression analyses of factors associated with the QoLA score.

Predictor	Univariate Regression Analysis				Multivariate Regression Analysis			
	Estimate	95% Confidence Interval		*p*	Estimate	95% Confidence Interval		*p*
		Lower	Upper			Lower	Upper	
Maternal age:								
20–29 years—Less than 20 years	17.3	−0.879	35.4	0.062	-	-	-	-
30–39 years—Less than 20 years	15.6	−2.311	33.6	0.087	-	-	-	-
More than 40 years—Less than 20 years	17.5	−0.616	35.6	0.058	-	-	-	-
Marital status:								
Divorced or separated—Married	−5.13	−14.1	3.85	0.262	-	-	-	-
Widow—Married	−18.46	−57.6	20.73	0.354	-	-	-	-
Occupational status:								
Non-employed—Employed	2.25	−3.11	7.61	0.409	-	-	-	-
Nationality:								
Non-Saudi—Saudi	−0.764	−10.7	9.13	0.879	-	-	-	-
Educational level:								
High school certificate—Less than high school education	−0.364	−9.15	8.42	0.935	-	-	-	-
Bachelor’s degree—Less than high school education	−3.368	−11.4	4.66	0.409	-	-	-	-
Postgraduate degree—Less than high school education	3.467	−9.21	16.14	0.59	-	-	-	-
A number of children:								
Two—One	1.99	−7.0699	11	0.665	-	-	-	-
Three—One	1.71	−7.2612	10.7	0.707	-	-	-	-
More than three—One	8.44	−0.0634	16.9	0.052	-	-	-	-
A number of autistic children:								
More than one—One	−7.81	−19.4	3.78	0.185	-	-	-	-
Family income:								
SAR 5000–10,000—Less than SAR 5000	8.53	1.63	15.4	0.016	4.83	−1.39	11.05	0.128
More than SAR 10,000—Less than SAR 5000	12.25	5.18	19.3	<0.001	8.05	1.65	14.44	0.014
Residency region:								
Western—Central	3.434	−6.71	13.58	0.505	-	-	-	-
Southern—Central	−8.778	−22.35	4.79	0.204	-	-	-	-
Eastern—Central	−5	−20.05	10.05	0.513	-	-	-	-
Northern—Central	−0.627	−8.86	7.61	0.881	-	-	-	-
MSPSS score mean	6.64	5.01	8.26	<0.001	6.29	4.65	7.93	<0.001

## Data Availability

The data supporting the findings of this study are available from the corresponding author upon reasonable request.
